# Phenotype with a side of genotype, please: Patients, parents and priorities in rare genetic disease

**DOI:** 10.1016/j.atg.2016.01.002

**Published:** 2016-02-01

**Authors:** Christy Collins

**Affiliations:** M-CM Network, Chatham, NY, United States

## Abstract

As the parent and caregiver of a child with an ultra-rare disease and advocate for others with the same condition, I discuss the importance of phenotyping in rare disease research. I emphasize the need for more clinical geneticists, deeper and more intentional integration of clinical genetics in complex patient care, and a greater appreciation of patients and families as an informational resource.

My six-year-old daughter has an extremely rare genetic condition with too many names. The one I use is M-CM, or macrocephaly-capillary malformation syndrome (Toriello and Mulliken, 2007).

M-CM was first delineated as a syndrome in two papers in 1997, one from the United Kingdom (Clayton-Smith et al., 1997) and one from the United States (Moore et al., 1997). The papers were written by clinical geneticists with dysmorphology expertise who had seen more than one patient and had identified a pattern of remarkable features. Prior to 2012, there were no known inherited cases, so the condition was considered to be sporadic. Several papers subsequently refined the description of the phenotype and suggested diagnostic criteria (Robertson et al., 2000, Franceschini et al., 2000, Wright et al., 2009, Martínez-Glez et al., 2010). In June 2012 mosaic *PIK3CA* mutations were identified as a genetic cause for M-CM (Rivière et al., 2012).

My daughter had a difficult and frightening start due to pleural effusion: she was intubated from birth to ten days of age. While still in the NICU she displayed a richness of observable phenotypic gifts that could have made her diagnosis fairly easy had anyone with appropriate skills been available to us. She was asymmetrical from her head to her toes, enormous at 11 lb, yet very weak. Her second and third toes were joined together on each foot, the majority of her body and part of her face were covered in reddish birthmarks, and there was a darker birthmark on her lower lip that gave her a Pierrot-doll appearance. Finally, she had a sweet button nose that does not run in our family.

I would gradually learn the clinical names for some of these features, names needed in order to find a diagnosis: hemihyperplasia, macrosomia, hypotonia, syndactyly, depressed nasal bridge. The red birthmarks have unreliable nomenclature, and some might argue that they should not be called birthmarks at all. Researchers seem to have settled on capillary malformations, which is a correct descriptor. But they have also been referred to as cutis marmorata telangiectatica congenita, hemangioma, and cutis marmorata. Some patients do indeed have some of these other vascular anomalies, but often the terms have been applied incorrectly.

Living in a Lyme-endemic area gave us an early introduction to the tendency to gravitate to the familiar. I had gotten sick in the middle of my pregnancy and was treated for Lyme disease as a precaution. The first theory about my daughter's condition was that it was congenital Lyme, the very existence of which is controversial. Infectious disease experts were contacted, tests were run, and this idea was dismissed before we left the NICU at 2.5 weeks.

We next visited a vascular anomalies team composed of surgeons and dermatologists. The extensive red birthmarks and asymmetry pointed to the appropriateness of this team. They confidently provided a diagnosis that had not been published in the medical literature but was based on their experience of seeing a high volume of patients with vascular anomalies. We were told that, in spite of her rough start, our daughter's issues would only be cosmetic. This team did not include neurologists, who might have recognized hypotonia and flagged important neurological concerns. It also did not include a geneticist, who might have seen the bigger picture.

This was a very difficult time for us, perhaps the most difficult of my life. My daughter had hip dysplasia and was so hypotonic and hypermobile that when the local orthopedist felt the way that her legs moved, he immediately told us that we needed more specialized care than he could provide. But because we had been told that our daughter's condition was strictly cosmetic, we couldn't convince our insurance company that she needed specialized orthopedic care.

Eventually, after a couple of months of making it my full-time job to obsessively search the internet, I found my daughter's diagnosis. I did not find it in a journal article or a diagnostic algorithm. It was in a comment of a blog kept by the parent of a baby who looked like my daughter. That comment led to another blog about a child who was an unmistakable match for her. I convinced my daughter's primary care doctor to order a brain MRI, which showed correlative brain findings. We eventually had the diagnosis confirmed by a local geneticist, and then by another geneticist who had some expertise in the condition. This was shortly before the discovery of *PIK3CA*'s involvement in M-CM.

When I look back on this period, knowing what I know now about genetic conditions and the characteristics of my daughter's syndrome, the lack of clinical genetics knowledge among other specialties and general pediatrics was stunning. My daughter had an observable phenotype that hit the bullseye for her diagnosis, but none of the professionals that we saw had the training to interpret the significance of her features that fell outside of their specialties, nor even to recognize that a geneticist might understand a constellation of physical signs and have some talent other than administering genetic tests. A vague order of “genetic testing” had been given by one of the NICU doctors, but nobody knew specifically what to test for — so the order was dropped.

This lack of access to clinical genetics is hardly unique to our story. In Facebook groups for characteristics that my daughter has (hypotonia and hemihyperplasia), undiagnosed people regularly share questions and confusion about whether their condition is a thing unto itself or part of a larger syndrome. Group participants suggest that they get a genetics referral, which has not been offered by their providers and which is sometimes met with resistance by their current primary care and/or specialist clinicians. Patients then come back and ask if a five- to six-month wait to see genetics is normal, and the support group participants reply that, unfortunately, it is.

The vast majority of these interactions between anxious parents and ill-equipped clinicians involve families seeking care for young children. Older patients born before the existence of a clinical description are less likely to come to a genetics clinic. In the M-CM population, for example, anyone over 18 years of age would have been born before a diagnosis existed. These patients seem to get diagnosed only if they have mysterious health problems and a caregiver decides it is worthwhile to engage in the difficult business of revisiting a diagnosis.

Just like any specialty, patient experiences with clinical genetics vary, and the role that the geneticist takes on in the patient's care is not consistent across providers. The reason for this is unclear to me, but I will share our experience. By the time we visited our first geneticist, who was local to us, I had heard about the experience of other families with M-CM. A small concentration of these people had seen the same couple of providers, who had cultivated some expertise in the syndrome and published their research and experiences. For these families, the geneticists followed their children over time and continued to consult on the condition long after the initial diagnosis, providing a sort of complex primary care. Besides the obvious benefits of ongoing specialized consultation for patients, the geneticists could also inform the patients about the most up-to-date findings related to the syndrome and suggest appropriate management.

My family was very excited for our first appointment with our local geneticist, thinking that we were going to meet someone who would provide ongoing guidance for this bewildering journey that we had found ourselves on. We were disappointed to find that the geneticist saw his role strictly as a diagnostician. He provided us with a one-page report with a few paragraphs about managing our daughter's condition for the rest of her life. We never saw him again.

We decided to make the 10-hour drive to see one of the geneticists who we knew had expertise in the syndrome. M-CM has highly variable severity across patients, and we wanted a sense of where our infant daughter fit along that spectrum. This geneticist spent several hours with us, thoroughly examined our daughter and reviewed her brain MRIs. He answered all of our questions as best he could — many of the answers were “we don't know,” which was informative in its own way. Shortly after we got home from the visit, a 17-page report arrived that covered every body system known to be affected by M-CM and suggested appropriate monitoring and management. Now we had a detailed document to provide to other specialists about a condition that they might see once in their careers, if ever.

The clinical geneticists who provide this level of attention, along with the ones who originally recognized the phenotypic pattern of the condition, are the heroes of our M-CM story. Phenotype is really what we care about as patients and parents. We want to know what to expect and the best way to manage the challenges. We especially want to connect with people who are facing the same specific issues as us in order to learn from their experiences.

Unfortunately, the geneticist that we traveled to now works in a specialty clinic and is unable to see M-CM patients. At the time, one other geneticist in the United States had a similar level of clinical experience and trust from the M-CM patient community, but that geneticist is also no longer available to patients. It is not unusual for rare disease patients to have a very limited number of clinical experts to turn to, and if one of these experts does not work out for any reason – such as distance, insurance coverage, availability, or even personality – patients are on their own.

Since the genetic cause of M-CM was identified in 2012, the M-CM literature has shifted from a focus on case studies with phenotypic and medical descriptions to one on genomics and genomic testing. While we recognize the importance of genomic science for reproductive planning and future therapeutics, from our family's perspective it is hard to get excited about this shift. The research that affects us most directly concerns outcomes, optimal care, and management based on the resources available to us *now*.

It turns out that M-CM shares mosaic activating mutations in *PIK3CA* with several other syndromes (Keppler-Noreuil et al., 2014), some of which have previously gotten more attention from vascular anomalies specialists than geneticists. These syndromes are now viewed as existing on a spectrum. The task for geneticists will be to untangle genotype–phenotype correlations across the distinct entities. Many patients have cycled through multiple diagnoses for these various conditions, so parent and patient advocates were connected and seeing overlap well before the genetic bases were identified.

This recognition is a testament to the significance of phenotype. Some patients along the *PIK3CA*-overgrowth spectrum express confusion when they are told that they may have another of these conditions instead of their current diagnosis. The only thing distinguishing the conditions right now is phenotype. It's hard for people who may land on the margins of a particular OMIM description to understand that a genetic test cannot currently provide more clarity than a thorough description of phenotype.

I believe that the best, fastest, and perhaps only way for these conditions to be understood is for patients to be seen in pediatric specialty clinics that 1) attract a relatively large volume of patients; and 2) strongly integrate clinical genetics into their practices. I suspect this may apply to many other conditions as well, and not necessarily just rare ones. For example, might obesity clinics discover phenotypic clusters with genetic underpinnings?

In the absence of information available in clinical settings, parents and patients go online to find others facing similar issues (Might and Wilsey, 2014). We naturally group ourselves according to syndrome characteristics, and our insights into the ways that the groups we belong to are affected is generally underappreciated and underutilized by doctors and researchers. Patient families are highly motivated and invested in building knowledge about their conditions; at the risk of immodesty, we do an incredible job of it with the tools available to us.

Sequencing genomes is easy. Phenotyping is hard. But if we have any hope of making progress in understanding M-CM and countless other rare conditions, researchers must leverage patient communities in order to do the hard part.

So how can we raise the stature of phenotype? In publications on my daughter's condition, the phenotype is usually no more than a short list of features, or the written-out words of the acronym used to name the condition. I read a paper recently with exquisitely detailed phenotypic descriptions for each individual but, as it so often is, this text was relegated to the supplement, i.e., it was not thought to be important enough to include in the main body of the paper.

As the Precision Medicine Initiative™ ramps up and defines itself, the problem of phenotyping poses a daunting challenge. Rare diseases make strong cases for developing phenotyping methods that can be married to the computational outcomes of genomic evaluation. This needs to be done without steamrolling the human interactions in the clinic that are required for effective patient care and research. Rare disease patient families will be eager and valuable partners in this effort if given the opportunity.

Given that our medical system currently seems unable to provide sufficient genetics professionals for patients to be seen in a timely manner, other front-line doctors will need better diagnostic and observational skills, and awareness of how the pieces of a diagnostic puzzle may lie outside of their specialty. It will be unfortunate if in the clinic, as in the literature, a shift toward genetic sequencing and analysis comes at the expense of skill and interest in clinical phenotyping.
